# Bacterial Community Legacy Effects Following the Agia Zoni II Oil-Spill, Greece

**DOI:** 10.3389/fmicb.2020.01706

**Published:** 2020-07-17

**Authors:** Gareth E. Thomas, Tom C. Cameron, Pablo Campo, Dave R. Clark, Frederic Coulon, Benjamin H. Gregson, Leanne J. Hepburn, Terry J. McGenity, Anastasia Miliou, Corinne Whitby, Boyd A. McKew

**Affiliations:** ^1^School of Life Sciences, University of Essex, Colchester, United Kingdom; ^2^School of Water, Energy and Environment, Cranfield University, Cranfield, United Kingdom; ^3^Institute for Analytics and Data Science, University of Essex, Wivenhoe Park, Essex, United Kingdom; ^4^Archipelagos Institute of Marine Conservation, Samos, Greece

**Keywords:** Mediterranean, hydrocarbons, Agia Zoni II, oil spill, *Alcanivorax*, *Cycloclasticus*, *Idiomarina*, Greece

## Abstract

In September 2017 the Agia Zoni II sank in the Saronic Gulf, Greece, releasing approximately 500 tonnes of heavy fuel oil, contaminating the Salamina and Athens coastlines. Effects of the spill, and remediation efforts, on sediment microbial communities were quantified over the following 7 months. Five days post-spill, the concentration of measured hydrocarbons within surface sediments of contaminated beaches was 1,093–3,773 μg g^–1^ dry sediment (91% alkanes and 9% polycyclic aromatic hydrocarbons), but measured hydrocarbons decreased rapidly after extensive clean-up operations. Bacterial genera known to contain oil-degrading species increased in abundance, including *Alcanivorax*, *Cycloclasticus*, *Oleibacter*, *Oleiphilus*, and *Thalassolituus*, and the species *Marinobacter hydrocarbonoclasticus* from approximately 0.02 to >32% (collectively) of the total bacterial community. Abundance of genera with known hydrocarbon-degraders then decreased 1 month after clean-up. However, a legacy effect was observed within the bacterial community, whereby *Alcanivorax* and *Cycloclasticus* persisted for several months after the oil spill in formerly contaminated sites. This study is the first to evaluate the effect of the Agia Zoni II oil-spill on microbial communities in an oligotrophic sea, where *in situ* oil-spill studies are rare. The results aid the advancement of post-spill monitoring models, which can predict the capability of environments to naturally attenuate oil.

## Introduction

**FIGURE 1:**
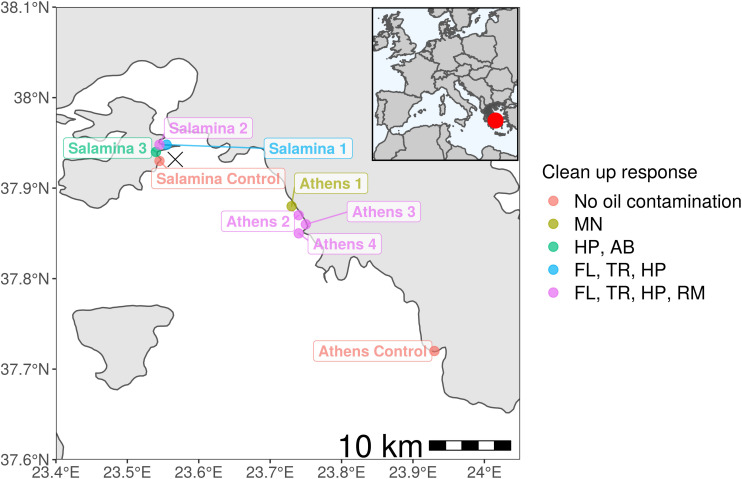
Sampling sites from the Agia Zoni II oil-spill (marked as an X where tanker sank) in Greece (inlet; dark gray; red dot highlights spill-site) and affected Athens Riviera and Salamina coastline. Abbreviations for clean-up response applied at oil-contaminated sample sites (Athens 1–4 and Salamina 1–3): MN (manual removal of tar balls), HP (high-powered washing, to remove oil from hard surfaces), AB (use of absorbents to collect floating oil from the water surface), FL (flushing of sediment with medium pressure water), TR (trenching, used in conjunction with flushing to collect oil), and RM (removal of coastal sediment, either washed and replaced or disposed in landfill).

On the 10th of September 2017, the Agia Zoni II tanker sank in the inner Saronic Gulf, Greece, releasing approximately 500 metric tonnes of heavy fuel oil into the waters and contaminating the surrounding coastlines (IOPC, 2017). The Hellenic Coast Guard deployed ∼600 m of oil boom out to sea within 8 h, which increased to ∼9 km of booms and absorbents in the following weeks. Despite this response, the oil had spread far from the spill site and impacted over 4 km of the Salamina coastline and over 25 km of the Athens Riviera including Glyfada and the port of Piraeus, due to a change in the wind direction (Figure 1). An extensive clean-up response was undertaken on all contaminated beaches, including manual removal of tar balls, flushing and trenching, high-powered washing, removal of sediments for either washing/replacement or disposal at landfill, and the use of absorbents (see Figure 1 and Supplementary Table S1 for sample location and specific treatment/action undertaken at each site). On the 30th November, 2017 the shipwreck was removed, and the clean-up operations ceased in February 2018. On the 28th April, 2018 the Greek government lifted maritime restrictions.

Oil spills dramatically alter marine microbial community composition, resulting in a decrease in species richness and diversity, in conjunction with selection for oil-degrading bacteria (Head et al., 2006; McGenity et al., 2012). Certain microbes can degrade a range of hydrocarbons found in crude oil and its derivatives, including obligate hydrocarbonoclastic bacteria (OHCB), which use hydrocarbons as an almost exclusive source of carbon and energy (Yakimov et al., 2007). While OHCB have been demonstrated to degrade other compounds in pure culture (Radwan et al., 2019; Zadjelovic et al., 2020), there is still limited evidence that the OHCB are competitive for non-hydrocarbon substrates in the environment. This is evidenced by the fact OHCB are typically present in extremely low numbers in uncontaminated environments but rapidly increase in abundance following oil-spills (Yakimov et al., 2004a; Atlas and Hazen, 2011; Acosta-González et al., 2015; Nogales and Bosch, 2019). Therefore, while it is evident that OHCB use a few other carbon and energy sources, for clarity we refer to them hereafter as “OHCB” to distinguish between these highly adapted and competitive hydrocarbon degraders and those more metabolically diverse bacteria, which can also degrade hydrocarbons. Since the deepwater horizon well blowout, there has been increased emphasis on understanding oil-spill microbial ecology, with much of the focus on deep sea oil plumes (Hazen et al., 2010; Mason et al., 2012; Redmond and Valentine, 2012), salt marsh sediments (Beazley et al., 2012), or benthic sediments (Mason et al., 2014). Though some studies have focused on coastal sediments (Kostka et al., 2011; Lamendella et al., 2014; Huettel et al., 2018) most were conducted either *ex situ* (Röling et al., 2002; Da Silva et al., 2009; Almeida et al., 2013) or retrospectively (De La Huz et al., 2005; Short et al., 2007; Boufadel et al., 2010), and represented a bias toward a single oil-spill event, deepwater horizon. Therefore, the *in situ* establishment and succession of specialized oil-degrading microbial taxa, in the Mediterranean immediately following an oil spill, are less well understood. This is especially true for coastal sediments in the Mediterranean (see Supplementary Table S2). Understanding how such ecosystems, and particularly oil-degrading microbial communities, respond to such events will allow for better post-spill monitoring (Kirby et al., 2018). Exposure of environments to oil can also lead to a long-lasting adaptation within the microbial community. This phenomenon of prior exposure can be important in determining the rate at which any subsequent hydrocarbon inputs may be biodegraded (Leahy and Colwell, 1990). Such knowledge will also assist in designing or improving models such as the Ecological Index of Hydrocarbon Exposure (EIHE) (Lozada et al., 2014) and inform future oil-spill response, such as indicating the thoroughness of clean-up efforts and identifying when tourist/fishing activities can recommence.

The Mediterranean is an oligotrophic sea with extremely low levels of phosphorus (Thingstad et al., 2005) and is virtually an enclosed basin with limited oceanic exchange. Phosphorous is a macronutrient that is vital for microbial growth and especially for hydrocarbon degradation (Seah and McFerran, 2016); and therefore, the availability of these nutrients in the presence of hydrocarbons is vital (Ron and Rosenberg, 2014). From 1977 to 2018 the Mediterranean has been subject to 989 recorded oil-pollution incidents (REMPEC, 2018). Additionally, the Mediterranean has been the location of three of the world’s top 20 largest tanker oil spills; including the tankers Haven (1991, 144,000 tonnes, Italy), Irenes Serenade (1980, 100,000 tonnes, Greece), and Independenta (1979, 95,000 tonnes, Turkey) (ITOPF, 2019). The majority of accidents resulting in oil-pollution occur in the Eastern Mediterranean, especially around Greece (Girin and Carpenter, 2019). The Mediterranean currently contains two refineries, two oil terminals, and three oil ports (REMPEC, 2018), with an additional 480 active shipping ports, 50% of which are in located around Greece and Italy (Xue et al., 2004). The Mediterranean is highly socio-politically complex with 21 countries bordering the sea, and future predictions indicate increased levels of oil and gas drilling in the region (Union et al., 2016). Oil pollution in the Mediterranean therefore represents a significant threat to the environment. Despite this, there remains a paucity of information on how oil pollution affects Mediterranean sediment microbial communities.

Our early sampling (5 days post spill) of the Agia Zoni II incident allowed a very rare opportunity to investigate the immediate impact on the microbial community and identify the earliest key colonizing oil-degrading bacteria from an oil spill that covered a large area of coastal sediment, in a region where fishing and tourism play a pivotal socio-economic role. We quantified hydrocarbon concentrations across seven contaminated and two uncontaminated sites 5 days post-spill and after remediation. Additionally, we determined oil-spill-induced changes in gene abundance, diversity, and composition of the sediment microbial communities using qPCR and high throughput sequencing of the 16S rRNA gene.

## Results

### Sediment Hydrocarbon Concentrations

**FIGURE 2:**
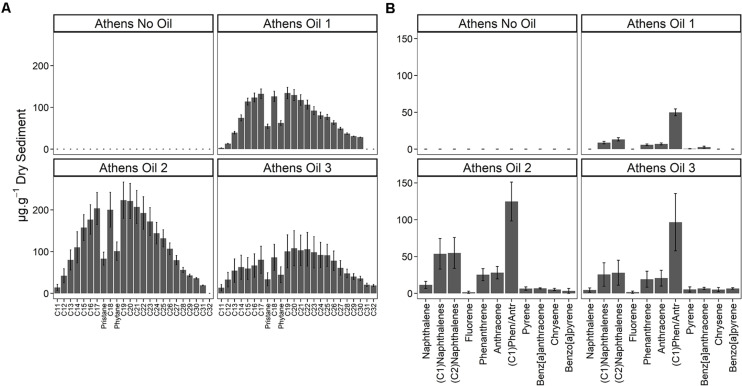
Hydrocarbon concentrations (mean ± SE, *n* = 3) of the three contaminated sites (Athens Oil 1-3) and the uncontaminated control site (Athens No Oil), September 2017. **(A)** *n*-alkanes from C11 to C32 and the branched alkanes pristane and phytane. **(B)** PAHs from 2-ring naphthalene to 5-ring benzo[*a*]pyrene. No samples were taken for Salamina in September 2017.

**FIGURE 3:**
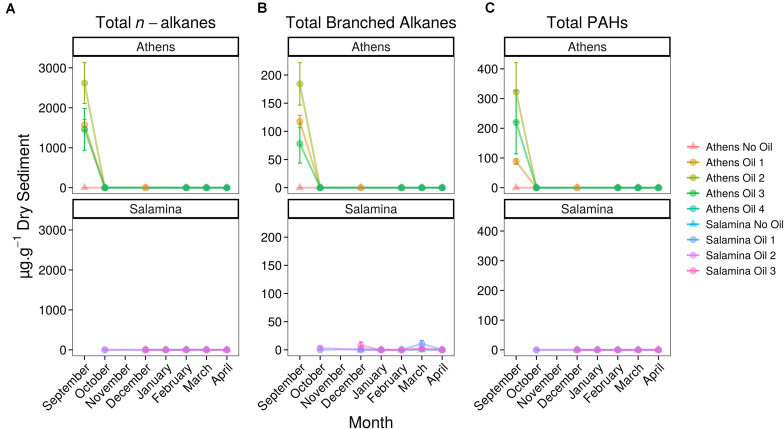
Concentrations (mean ± SE, *n* = 3) of hydrocarbons in control (Suffix “No Oil”) and contaminated (Suffix “Oil”) sediments along the Athens Riviera and Salamina coastline, sampled from September 2017 to April 2018. **(A)** Total *n*-alkanes, **(B)** total branched alkanes (pristane and phytane), and **(C)** total PAHs. No samples were collected for all Salamina sites in September 2017, “Salamina No Oil” in October 2017, or all Salamina and Athens sites in November 2017.

Five days after the oil spill, the surface sediments at three sample sites on the Athens Riviera were visibly contaminated by hydrocarbons (Figure 2). The concentrations of the aliphatic hydrocarbons fraction including *n*-alkanes from undecane (C_11_) to dotriacontane (C_32_) and the branched alkanes (pristane and phytane) ranged from 1,536 ± 557 to 2,803 ± 549 μg g^–1^ dry sediment (Figure 2). The concentrations of the aromatic fraction, containing the 2–5 fused-ring polycyclic aromatic hydrocarbons (PAHs), including methylnaphthalenes, dimethylnapthalenes, methylphenanthrenes, and methylanthracenes ranged from 88.20 ± 13.50 to 332 ± 99 μg g^–1^ dry sediment. One-month post-spill, the concentration of total *n*-alkanes, branched alkanes, and PAHs reduced to almost undetectable levels at both the Athens Riviera and Salamina sites (Figure 3). Additionally, we found no significant differences in phosphate, nitrate, and ammonium concentrations between contaminated sites and uncontaminated control sites when compared to coastal water samples (Supplementary Figure S1).

### Effects of the Oil-Spill on Sediment Microbial Community Composition and Abundance

In order to evaluate any effect that the oil-spill had on microbial community composition or abundance, 16S rRNA bacterial and archaeal genes were analyzed by qPCR and amplicon library sequencing [see operational taxonomic units (OTU) tables in Supplementary Tables S3, S4]. High-throughput 16S rRNA gene sequencing resulted in an average of 55,591 (range 8,093–209,105) and 49,983 (range 3,822–196,697) sequence reads for bacteria and archaea, respectively. Five days post-spill, the mean bacterial 16S rRNA gene abundance (1.6 × 10^8^ copies g^–1^ dry sediment) in Athens contaminated sediments was ∼2.5 fold greater, but not statistically significant, than in the uncontaminated control sediments (Supplementary Figure S2A). Archaeal 16S rRNA gene abundance was typically two orders of magnitude lower than the Bacteria (Supplementary Figure S2B) and no significant changes were observed in archaeal 16S rRNA gene abundance in response to the oil spill. Simpson Diversity Index analysis of archaeal and bacterial 16S rRNA gene amplicon libraries revealed no significant difference between contaminated (average across all samples, archaea 0.35 and bacteria 0.99) and uncontaminated (archaea 0.26 and bacteria 0.99) sites, at any month. Archaeal community analysis revealed a high relative abundance of *Candidatus* Giganthauma and *Nitrosopumilus*, though no significant differences were observed between contaminated and uncontaminated sediment samples. This suggests archaea did not play a significant role in biodegradation of, and were unaffected by, the oil (Supplementary Figure S3).

**FIGURE 4:**
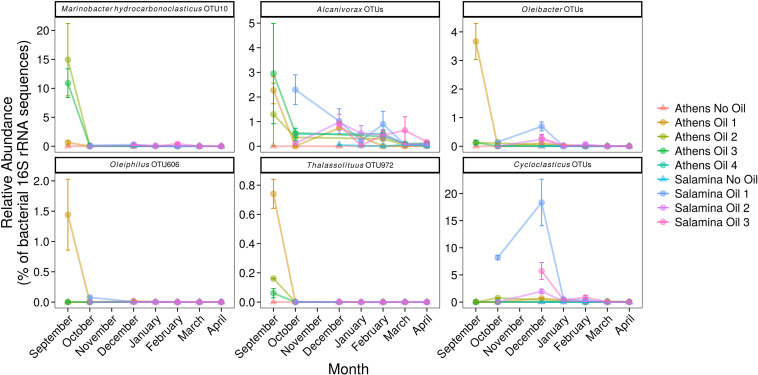
Relative abundance (% of the bacterial community; mean ± SE, *n* = 3) of 16S rRNA gene OTUs assigned to putative obligate hydrocarbonoclastic bacteria (including OTU-10 which has a 100% identity match to *M. hydrocarbonoclasticus*) in sediments from control (Suffix “No Oil”) and contaminated (Suffix “Oil”) sites along the Athens Riviera and Salamina coastline, from September 2017 to April 2018. No samples were collected for all Salamina sites in September 2017, “Salamina No Oil” in October 2017, or all Salamina and Athens sites in November 2017. Bacteria and Archaea OTU tables are available in Supplementary Tables S3, S4.

**FIGURE 5:**
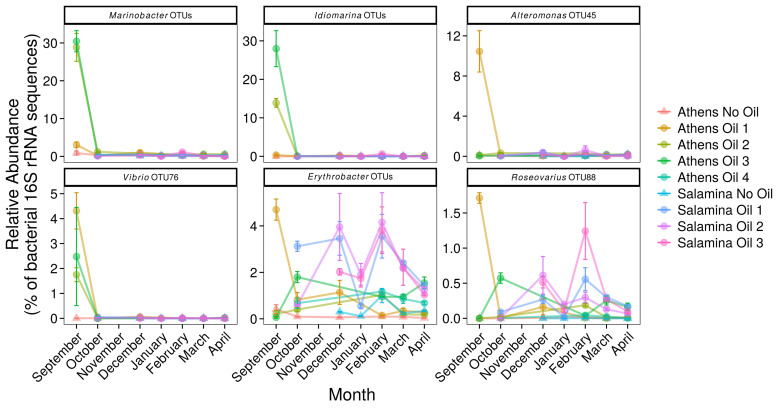
Relative abundance (% of the bacterial community; mean ± SE, *n* = 3) of 16S rRNA gene OTUs assigned to Bacteria associated with oil-degradation, in sediments from control (Suffix “No Oil”) and contaminated (Suffix “Oil”) sites along the Athens Riviera and Salamina coastline, from September 2017 to April 2018. No samples were collected for all Salamina sites in September 2017, “Salamina No Oil” in October 2017, or all Salamina and Athens sites in November 2017. Bacteria and archaea OTU tables are available in Supplementary Tables S3, S4.

Operational taxonomic units affiliated to numerous OHCB in contaminated sites 5 days post-spill were significantly more relatively abundant in comparison to uncontaminated control sites. These changes included higher relative abundance of members of *Marinobacter*, closely related to *M. hydrocarbonoclasticus*, *Alcanivorax* spp., *Oleibacter* spp., *Oleiphilus* spp., and *Thalassolituus* spp. (Figure 4) in comparison to uncontaminated sites. In October and December higher relative abundance of *Cycloclasticus*, a known PAH-degrading genus (Dyksterhouse et al., 1995) was also observed in the contaminated site “Salamina Oil 1.” There was also significantly higher relative abundance of sequences affiliated to more catabolically versatile genera or species (i.e., those that use a wider range of substrates than the OHCB whose substrate range in the environment is typically restricted to hydrocarbons) in comparison to uncontaminated sites. These genera have been shown to contain representatives that can degrade hydrocarbons; including *Marinobacter* spp., *Idiomarina* spp., *Alteromonas* spp., *Vibrio* spp., *Erythrobacter* spp., and *Roseovarius* spp. (Figure 5; see Supplementary Table S2). Dissimilarities in bacterial community composition were evident between uncontaminated and contaminated sites in September 2017 immediately after the oil-spill. However, from October 2017 onward the bacterial community composition at the oiled sites become increasingly similar to as those found at the uncontaminated sites (Supplementary Figure S4).

**FIGURE 6:**
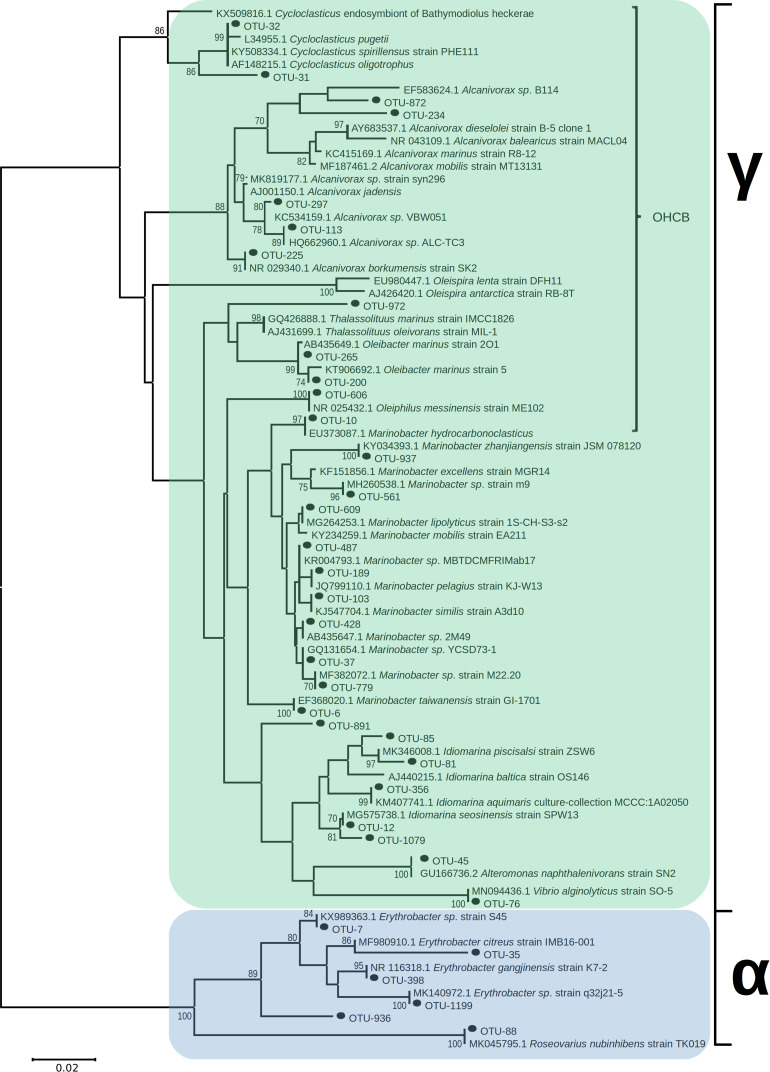
Unrooted neighbor-joining phylogeny of 16S rRNA bacterial OTUs, in sediments from contaminated sites along the Athens Riviera and Salamina coastline, from September 2017 to April 2018, aligned with known hydrocarbon-degrading bacteria and closest relatives; bootstrap values >70 displayed (1,000 iterations). Evolutionary distances computed by Maximum Composite Likelihood protocol (using the Tamura-Nei model, Tamura and Nei, 1993), sum of branch length = 1.15. Analysis involved 82 nucleotide sequences (36 OTUs) with a total of 258 positions in the final dataset. Evolutionary analyses were conducted in MEGA7. Bacteria and archaea OTU tables are available in Supplementary Tables S3, S4.

Several of the OTUs that were significantly more relatively abundant at contaminated versus uncontaminated sites were affiliated to OHCB and specifically to known alkane degraders. For example, OTU-10 (100% 16S rRNA sequence identity to *Marinobacter hydrocarbonoclasticus*; Figures 4, 6), had significantly higher relative abundance at some contaminated sites (coef. = 10.88, *z* = 18.13, *p* < 0.001) compared to uncontaminated sites. Five days post-spill, OTU-10, represented 15 and 11% at “Athens Oil 2 and 3,” respectively, but sharply decreased in relative abundance to virtually undetectable levels from October onward. The increase in the relative abundance of *M. hydrocarbonoclasticus* represented an increase of 2.14 × 10^7^ 16S rRNA gene copies assigned to *M. hydrocarbonoclasticus* compared to uncontaminated Athens sediments. Five OTUs assigned to the OHCB genus *Alcanivorax* (Figures 4, 6) were significantly more relatively abundant in all contaminated sites (September 2.19% ± 1.52%; coef. = 2.28, *z* = 6.40, *p* < 0.001) compared to uncontaminated sites where it was generally undetectable. From October the relative abundance of *Alcanivorax* OTUs steadily decreased in the contaminated sites, though always remained at least 5- to 297-fold greater than the uncontaminated control sites, where they were often undetectable. Similarly, OTUs from the genus *Oleibacter* (OTU-200 and OTU-265) and OTU-606 from *Oleiphilus* (Figures 4, 6) were significantly more abundant in “Athens Oil 1” immediately after the oil-spill in September (coef. = 1.44, *z* = 13.35, *p* < 0.001) from uncontaminated control levels (3.67 ± 0.89% and 1.44 ± 0.83%, respectively). However, in April the relative abundance of both genera decreased to levels below 0.01%. Finally, OTU-972 assigned to *Thalassolituus* (Figures 4, 6), a genus containing species that can degrade a wide range of alkanes (Yakimov et al., 2004b; Gregson et al., 2018), was significantly more relatively abundant in sediments from contaminated sites in September (0.74%; coef. = 0.16, *z* = 7.52, *p* < 0.001) compared to uncontaminated control sediments. The relative abundance of *Thalassolituus* decreased in the months thereafter to undetectable levels. The increase in the relative abundance of *Thalassolituus* represented an increase of 1.22 × 10^6^ 16S rRNA gene copies compared to uncontaminated Athens sediments, even though *Thalassolituus* was the least abundant OCHB genus.

No known PAH-degrading OHCB were detected five days post-spill when high PAH concentrations were measured. However, OTUs from more metabolically diverse genera (which have been demonstrated to degrade PAHs; see Supplementary Table S2) were observed in significantly higher relative abundance (coef. = 1.75, *z* = 6.06, *p* < 0.001) compared to uncontaminated levels, including *Alteromonas* spp. (10.46 ± 2.91% at “Athens Oil 1”), *Idiomarina* spp. (13.96 ± 11.75% at “Athens Oil 2 and 3”), and *Vibrio* spp. (2.09 ± 2.03% across “Athens Oil 1, 2, and 3”). OTU-31 and OTU-32, assigned to the PAH-degrading genus *Cycloclasticus* (Figures 4, 6), had significantly greater relative abundance in some contaminated locations compared with uncontaminated control sites. For example, in October and December they contributed up to 19% of relative abundance at “Salamina Oil 1” (coef. = 18.31, *z* = 19.21, *p* < 0.001), sharply reducing thereafter.

Ten OTUs belonging to the genus *Marinobacter* (excluding sequences assigned to the OHCB species *M. hydrocarbonoclasticus*) (Figures 5, 6), were significantly more relatively abundant in sediments taken in September from “Athens Oil 2 and 3” (30%; coef. = 27.94, *z* = 43.21, *p* < 0.001) relative to the uncontaminated sites, decreasing sharply from October. Similarly, five OTUs assigned to the genus *Idiomarina* (Figures 5, 6) were also significantly greater in relative abundance (coef. = 13.88, *z* = 23.40, *p* < 0.001), at 14 and 28% from sediments in “Athens Oil 2 and 3,” respectively, decreasing from October onward. OTU-45 assigned to the genus *Alteromonas* and OTU-76 assigned to the genus *Vibrio* (Figures 5, 6) significantly increased in relative abundance (coef. = 10.37, *z* = 26.11, *p* < 0.001), from uncontaminated control levels, particularly at contaminated “Athens Oil 1” to approximately 10 and 3% respectively, before sharply decreasing from October onward.

Two genera from the class Alphaproteobacteria also increased following oil contamination, which included five OTUs assigned the genus *Erythrobacter* and OTU-88 assigned to the species *Roseovarius* spp. (Figures 5, 6), which were significantly higher in relative abundance at contaminated sites from September to April, relative to levels at the uncontaminated sites.

Five days after the oil spill in September there was a strong positive correlation (*R*^2^ = 0.80, *p* < 0.01) between the relative abundance of 16S rRNA sequences assigned to hydrocarbon-degrading bacteria (all OTUs displayed in Figure 6) and the concentration of measured hydrocarbons. In addition, the EIHE, which quantifies the proportion of the bacterial community with hydrocarbon bioremediation potential on a scale of 0–1, whereby 1 represents 100% (Lozada et al., 2014), was calculated at 0.52 ± 0.14 in September. This EIHE result was significantly (*p* < 0.05) higher than the control site at 0.30 ± 0.11. At the genus level, some correlative relationships between bacterial genera containing hydrocarbon-degraders were observed. OTUs from the genera *Marinobacter* and *Idiomarina* typically co-occurred and were significantly correlated (*R*^2^ = 0.80, *p* < 0.05). A difference in the bacterial community composition occurred at different contaminated sites, despite the presence of similar hydrocarbons. For example, at “Athens Oil 2 and 3,” when *Marinobacter* and *Idiomarina* were in high relative abundance (collectively 46%) *Alteromonas*, *Oleibacter*, *Oleiphilus*, and *Thalassolituus* had low relative abundance of approximately 0.64%, collectively. In contrast, when *Marinobacter* and *Idiomarina* were in low relative abundance (collectively 4% at “Athens Oil 1” in September) *Alteromonas*, *Oleibacter*, *Oleiphilus*, and *Thalassolituus* had a relative abundance of approximately 16%, collectively, at the same site in September. The full correlation analysis showing the co-occurrence of taxa is provided in Supplementary Figure S5.

## Discussion

### Sediment Hydrocarbon Concentrations and Clean-Up Operations

Five days after the oil spill, GC-MS analysis revealed extensive oil contamination in the surface sediments at multiple beaches along the Athens Riviera. This contamination occurred due to rapid transfer of the oil from the water onto the coastal sediments within the first few days of the spill, due to sustained prevailing winds (Parinos et al., 2019). The level of total measured hydrocarbons, 2,158 ± 800 μg g^–1^ dry sediment, was similar to that observed from surface sediments in the Exxon Valdez oil-spill, 4,636 ± 1,628 μg g^–1^ sediment (Bragg et al., 1994). However, Agia Zoni II hydrocarbon sediment concentrations were much lower than that observed in buried sediments in Pensacola Beach, Florida (11,000 μg g^–1^ sediment total petroleum hydrocarbons) which was impacted by the Macondo oil spill (Huettel et al., 2018). Unbranched alkanes and PAHs from sediments after the Agia Zoni II oil spill were undetected from October 2017 onward; though some branched alkanes persisted at low concentrations (primarily at contaminated Salamina sites, Oct–Apr 7.31 ± 7.04 μg g^–1^ sediment).

The near complete reduction of hydrocarbons to undetectable levels, from October 2017 onward, is in line with measurements taken by the Hellenic Centre for Marine Research (HCMR) (Parinos et al., 2019) and is indicative of efficient physical removal and other clean-up operations, which occurred immediately after the oil-spill incident and continued through to February 2018. The preferential removal of the more readily degradable linear *n*-alkanes, as indicated by the presence of branched alkanes and no measurable *n*-alkanes or PAHs from October 2017 onward, would suggest biodegradation was occurring. The extent to which the removal of hydrocarbons can be attributed to either biodegradation, natural processes, or remediation efforts cannot be determined from our data. However, hydrocarbon data from Parinos et al. (2019) suggested the early onset of biodegradation, which was sustained for 85 days post-oil-spill, especially amongst phenanthrenes.

While absorbents, flushing, and sediment washing were used, a large proportion of the clean-up operations at the study sites consisted of direct sediment removal (whether contaminated or not) (International Oil Pollution Compensation Funds, 2018). Though effective in the removal of oil (Dave and Ghaly, 2011), the bulk removal of coastal sediment can be environmentally damaging (Owens, 1972; Gundlach and Hayes, 1978; Petersen et al., 2002), and, has economic and environmental costs associated with disposal, often to landfill sites. In the case of Agia Zoni II, some sediment washing and replacement occurred, but these responses were not adopted to the same degree as removal, likely due to the need for a rapid clean up to minimize the economic impact from loss of tourism.

### Ecology of Obligate Hydrocarbonoclastic Bacteria

An oil-spill perturbation is indicative of a discrete short term disturbance event (Shade et al., 2012), whereby changes in microbial community are largely trait driven, by the ability of individual species to occupy available niches due to hydrocarbon-degradation processes and pressures. This often manifests as a large increase in the absolute abundance of Bacteria, due to selection for hydrocarbon-degrading bacteria (Head et al., 2006), which was observed here with a ∼2.5-fold increase, but not significant, in the absolute abundance of the bacterial 16S rRNA gene immediately following the oil-spill. In particular, there was significantly greater relative abundance of genera with known hydrocarbon-degrading bacteria at contaminated sites in comparison to uncontaminated sites. These notable increases, along with a history of hydrocarbon pollution in the Saronic Gulf, indicates an indigenous community of hydrocarbon-degrading bacteria, albeit at mostly very low or undetectable levels, allowing for rapid colonization of oiled sediments and growth. Hydrocarbons within sediments five days after the oil spill supported the growth of OHCB, which rapidly became dominant, and were almost undetectable in uncontaminated sediments.

*Alcanivorax* is an obligate degrader of branched- and *n*-alkanes (Yakimov et al., 1998), although it has been shown to enhance degradation of PAHs (McKew et al., 2007). *Alcanivorax* is ubiquitous in the marine environment and is observed in a variety of hydrocarbon polluted environments (see Supplementary Table S2). In agreement with the results presented here, *Alcanivorax* is often observed to become abundant early on in coastal beach sediments and decrease in abundance as *n*-alkanes are removed from the environment (Head et al., 2006; Kostka et al., 2011; Rodriguez-R et al., 2015). *Alcanivorax* persisted for many months after hydrocarbons (unbranched alkanes and PAHs) were undetected within the environment. This legacy effect could be caused by several processes, including the use of wax esters (Rontani, 2010) or polyhydroxyalkanoates (Możejko-Ciesielska and Kiewisz, 2016) as a carbon and energy store for use when nutrient availability (e.g., N & P) is more favorable for cellular growth. Alternatively, this persistence could be due to the low levels of branched alkanes, such as pristane and phytane, observed within the sediments in the months following September. Unlike most OHCB, certain species of *Alcanivorax* can use branched alkanes, for example, *A. borkumensis* SK2T (Yakimov et al., 1998; Gregson et al., 2019), *A. dieselolei* B-5 (Liu et al., 2011), and *A. hongdengensis* A-11-3 (Wang and Shao, 2012).

Other alkane-degrading OHCB genera detected included *Oleibacter* (Teramoto et al., 2011), *Oleiphilus* (Golyshin et al., 2002), and *Thalassolituus* (Yakimov et al., 2004b; Gregson et al., 2018); though their abundance was primarily restricted to “Athens Oil 1” sediments. *Oleibacter*, *Oleiphilus*, and *Thalassolituus* have all been observed to increase in abundance in oil-contaminated marine environments, though until now evidence is lacking for detection in coastal sandy sediments (see Supplementary Table S2). Previous studies of oil-contaminated sediment (Coulon et al., 2012) and seawater (Teramoto et al., 2013) observed *Oleibacter* and *Alcanivorax* in similar abundances, in line with our observations. However, in seawater-based laboratory experiments *Thalassolituus* has been observed to outcompete other alkane-degraders such as *Alcanivorax* (McKew et al., 2007), through its use of medium- and long-chained alkanes (Gregson et al., 2019); this was not observed in the sediment samples collected from the Agia Zoni II oil-spill, whereby *Alcanivorax* was in greater abundance.

*Cycloclasticus* is a PAH-degrading genus (Dyksterhouse et al., 1995), apart from one lineage that degrades very short-chained alkanes (symbiont of *Bathymodiolus*, undetected in this study; Rubin-Blum et al., 2017). *Cycloclasticus*, which has been observed to grow in many marine environments including sandy coastal sediments (see Supplementary Table S2), was mostly undetected in sediments from Athens, but was observed in high abundance from October through December in Salamina sediments (note that no Salamina samples were taken in September). As found with *Alcanivorax*, *Cycloclasticus* demonstrated an oil-legacy effect, persisting in the sediments for several months after PAH contamination was undetectable. Once again this could be due to carbon and energy storage, or the persistence of trace amounts of high molecular weight PAHs. Alternatively, hydrocarbons can be undetected through analytical methods such as GC-MS (McKenna et al., 2013) when weathered hydrocarbons from an oil spill undergo transformation into oxygenated hydrocarbons (though currently there is no evidence that *Cycloclasticus* is able to degrade oxygenated hydrocarbons) (Kiruri et al., 2013), which can persist for years within oil/sand aggregates (Aeppli et al., 2018).

### Ecology of Metabolically Versatile Oil-Degrading Bacteria and Microbial Interactions

The specialist PAH degrader, *Cycloclasticus*, was mostly undetected in sediments collected in September from Athens, possibly because it was outcompeted by more metabolically versatile bacteria that can also degrade PAHs. Potential candidates for this PAH degradation are the genera *Idiomarina* [including OTUs most closely matched to PAH degraders *I. seosinensis* (Yuan et al., 2015; Gomes et al., 2018) (100% similarity) and *I. piscisalsi* (Nzila et al., 2018) (98.83% similarity)] and *Alteromonas* [including an OTU most closely matched to PAH degrading *A. naphthalenivorans* (Jin et al., 2011) (100% similarity)]. *Idiomarina* and *Alteromonas* can use a range of substrates for growth, including PAHs, and in this region (Athens) may have outcompeted *Cycloclasticus*, in contrast to the latter months in Salamina where *Cycloclasticus* dominated. Given the dominance of *Cycloclasticus* in October/December in Salamina, it could be speculated that this genus was also dominant in September, in contrast to Athens where more metabolically versatile bacteria became established. Alternatively, differences in the clean-up response may partly account for the observed differences in *Cycloclasticus* relative abundance, as, for example, the remediation process of sediment removal did not occur at “Salamina Oil 1 and 3” (see Figure 1 and Supplementary Table S1), where an increased relative abundance of *Cycloclasticus* occurred. This is also the case for sediments from “Athens Oil 1” where the presence of *Cycloclasticus* was observed, but not in “Athens Oil 2 and 3” sediments.

*Idiomarina* and *Alteromonas* have been found in a range of oil-contaminated environments, including sandy coastal sediments (see Supplementary Table S2). In this study it was observed that while both these genera increased in abundance in contaminated sites compared to uncontaminated sites, they did not co-occur. *Idiomarina* did, however, positively correlate with the genus *Marinobacter*; including OTU-10 most closely related to *M. hydrocarbonoclasticus*, which has been shown to degrade a range of alkanes (Gauthier et al., 1992). The co-occurrence of *Idiomarina* and *Marinobacter* was most notable between sediments from “Athens Oil 1” and “Athens Oil 2 and 3” and was to the competitive exclusion of other genera with known oil-degrading bacteria, including: *Alteromonas*, *Erythrobacter*, *Oleibacter*, *Oleiphilus*, and *Roseovarius*, which co-occurred. *Alteromonas* and *Oleibacter* have been recorded previously to co-occur (Neethu et al., 2019). The dominance of *Idiomarina* and *Marinobacter*, at “Athens Oil 2 and 3,” could potentially be due to indigenous species having a priority effect, providing a competitive advantage, or potentially due to source point pollution events. Alternatively, the absence of *M. hydrocarbonoclasticus* and *Idiomarina* from “Athens Oil 1” sediments could be due to the different remediation efforts at this site, where visible oil contamination was not as evident and thus only manual removal of tar balls occurred (see Figure 1 and Supplementary Table S1). The positive relationship between *Marinobacter* and *Idiomarina* has been observed before, including in oil-sludge samples (Albokari et al., 2015) and in sandy coastal sediments from the Macondo oil-spill where these two genera dominated (Joye et al., 2014). Additionally, Joye et al. (2014) observed that *Alcanivorax* was able to co-occur with *Marinobacter* and *Idiomarina*, as also observed in sediments from this study.

This study also demonstrates the ability of *Vibrio* (which is a genus that contains species that can degrade alkanes (Al-Awadhi et al., 2012; Imron and Titah, 2018) and PAHs (Melcher et al., 2002) to grow in several oil-contaminated environments; see Supplementary Table S2) to also avoid competitive-exclusion by *Marinobacter* and *Idiomarina*. This finding could indicate the ability of certain *Vibrio* to degrade a wide range of hydrocarbons, potentially including branched alkanes, like *Alcanivorax*, which may indicate why these genera are unaffected by the prevailing dominance of *Marinobacter* and *Idiomarina*. Indeed, certain species of *Vibrio* are known to attach to algal cells (Kumazawa et al., 1991; Hood and Winter, 1997) and many algae are known to produce branched alkanes (Binark et al., 2000) so perhaps there is a phototrophic–heterotroph interaction (McGenity et al., 2012) through the utilization of branched alkanes by *Vibrio*. However, increased relative abundance in *Vibrio* spp. was not matched with increased branched alkane concentration in Salamina, and therefore this remains unverified. This co-occurrence could also occur due to *Vibrio* using PAHs.

Lastly, species within the genera *Erythrobacter* and *Roseovarius* can degrade a range of hydrocarbons and grow in oil-contaminated marine environments, including sandy coastal sediments (see Supplementary Table S2). *Erythrobacter* and *Roseovarius* maintained consistently higher abundances at the previously oiled sites in comparison to uncontaminated sites. Suggesting that once the genera have established a presence within the community, through the utilization of hydrocarbons, they are able to maintain this even in the absence of such carbon and energy sources.

### Are Archaea Affected by Oil-Contamination Within Coastal Sediments?

In agreement with many studies, archaeal abundance and diversity was mostly unaffected in the sediment samples taken from after the Agia Zoni II oil-spill (Redmond and Valentine, 2012; Urakawa et al., 2012; King et al., 2015; Sanni et al., 2015). Archaea are not commonly considered as hydrocarbon-degraders, apart from certain *Halobacteria* (McGenity, 2010; Oren, 2017) which typically do not prevail in coastal seawater (exception herein being the genera *Haladaptatus* and *Halogranum*). There is growing evidence that growth of nitrifying-archaea, especially from the genus *Nitrosopumilus*, is inhibited by crude oil (Urakawa et al., 2012, 2019), though some studies do not show any significant inhibition by oil on archaeal populations (Newell et al., 2014; Bernhard et al., 2016). In this study, while there were some between-site differences in the relative abundance of OTUs assigned to *Nitrosopumilus* these were not significantly different. By quantifying archaeal species which are sensitive to oil-spill disturbances, post-oil-spill monitoring models can be made more efficient by including them as sentinel species (Kirby et al., 2018).

### Post-Oil-Spill Monitoring of Microbial Communities

The ability of those responding to an oil-spill (oil-spill responders, Government bodies, local authorities, oil-industries, NGOs) to evaluate the efficiency of clean-up operations, and thus provide guidance on future risk-based scenarios, is a vital endeavor to protect the environment and reduce socioeconomic impact. This has been recognized across the globe, and in the United Kingdom the cross-government initiative “PREMIAM” was published to this accord (Kirby et al., 2018). Understanding how microbes respond to such perturbations can assist to this regard and has the potential to be incorporated into tools such as the Spill Impact Mitigation Assessment (IPIECA and IOGP, 2017). Combining the data from both hydrocarbon analysis, microbial ecology, and other environmental sources, a more detailed and holistic understanding can be revealed. Microbial community analysis can highlight whether biodegradation may be taking place and the likelihood of the environment to naturally attenuate the oil-spill. Nutrient concentration analysis would reveal whether the environment is nutrient limited and should therefore be supplemented with fertilizer. Hydrocarbon analysis can detect the proportion of light and heavy molecules that could indicate the need for additional remediation operations.

Using microbial ecology as a post-oil-spill monitoring tool is still in its infancy, however, models and indices to assist in this effort are available. Lozada et al. (2014) published an “EIHE,” whereby hydrocarbon contamination was assumed based on the relative abundance of genera known to contain hydrocarbon-degrading bacteria. The simplicity of this model is both a strength and a weakness. It is simple enough that it can easily be adapted as oil-spill microbial knowledge progresses, but it is too simple to fully establish hydrocarbon exposure. For example, in this study our uncontaminated control sites had an EIHE index of 0.30 ± 0.11 (on a scale of 0–1), despite no hydrocarbons being detected. This is due to the model including all species within functionally diverse bacterial genera such as *Pseudomonas* which includes >200 different species, of which only a small minority are known to degrade hydrocarbons. The EIHE could be improved further by analyzing microbial community composition at a finer detail, perhaps at a species level, though this would require a tool to phylogenetically match OTUs to known oil-degrading species. Additionally, the inclusion of sentinel microbes, those that are adversely affected by oil-contamination (i.e., ammonia-oxidizing archaea; Urakawa et al., 2012, 2019), would also add comprehension to the index. By advancing the knowledge of oil-spill microbial ecology, understanding the genetics and metabolic capabilities of all hydrocarbon-degrading bacteria and their microbial community interactions, and then combining this with hydrocarbon and environmental data, models such as the EIHE can be improved.

### Experimental Procedures

#### Sampling Locations and Schedule

Sediments were surveyed at both the surface (0–5 cm) and deeper depths (down to approximately 60 cm) on initial and subsequent site visits. However, only surface sediments were observed to be oil contaminated, so all analyses were focused on this upper layer. Samples were collected, in triplicate, in sterile 50 ml sterile polypropylene containers from control (uncontaminated) and contaminated (oil contaminated) sites along the Athens Riviera and Salamina Island coast, Greece (see Figure 1 and Supplementary Table S1). Sediments were randomly sampled (*n* = 3) across the distance of each beach. Additionally, coastal seawater samples were collected (we were unable to obtain seawater samples from Athens or Salamina for September 2017) for nutrient analysis [ammonium (NH_4_^+^), phosphate (PO_4_^3–^), silicate (SiO_2_^–^), nitrate (NO_3_^–^), and nitrite (NO_2_^–^)] using a SEAL Analytical AA3 HR AutoAnalyzer tandem JASCO FP-2020 Plus fluorescence detector. Samples were collected in September 2017 5 days after the oil-spill, when oiling of sediment was visible, at the Athens sites. However, we were unable to obtain sediment samples from all Salamina site for September 2017. Thereafter, samples were collected over a 7-month period (except for October when no samples for “Salamina No Oil” were collected and November when no samples for all site were collected). Samples were immediately frozen on collection and stored at −20°C.

#### Hydrocarbon Degradation (GC-MS)

Hydrocarbons were extracted from thawed sediments. Samples (2 g) were dried with 2 g of anhydrous sodium (Na_2_SO_4_). Dried samples were extracted with 12 ml of hexane:dichloromethane (50:50) in 20 ml vials with Teflon-lined screw caps by horizontal shaking at 150 oscillations per min over 16 h and finally sonicated for 30 min at 20°C. After centrifugation 12,000 × *g* for 5 min, extracts were cleaned on Florisil^§^ columns by elution with hexane. Extracts were transferred in conical tubes and evaporated to 0.7 ml over an ice bath to minimize loss of light PAHs. Deuterated alkanes (nonadecane C_19_d_40_ and triacontane C_30_d_62_ at 10 μg ml^–1^) and PAH (naphthalene-d_8_ and anthrancene-d_10_ at 10 μg ml^–1^) internal standards were added to each sample and quantification was performed on an Agilent 7890A Gas Chromatography system coupled with a Turbomass Gold Mass Spectrometer with Triple-Axis detector, operating at 70 eV in positive ion mode, using conditions as previously described by Coulon et al. (2007). External multilevel calibrations were carried out using alkanes [Standard Solution (C_8_–C_40_); Sigma], methylated-PAHs (1-methylnapthalene, 2-methylanthracene, and 9,10-dimethylanthracene; Sigma), and PAH (QTM PAH Mix; Sigma) standards, the concentrations of which ranged from 1.125 to 18 μg ml^–1^. For quality control, a calibration standard (10 μg ml^–1^) and a blank were analyed every 10 samples. We quantified alkanes between C_10_ and C_36_ including pristane and phytane and the following PAHs: naphthalene; all isomers of methyl-, dimethyl- and trimethyl-naphthalenes; acenaphthylene; acenaphthene; fluorene; phenanthrene; all isomers of methyl- and dimethyl-phenanthrenes/anthracenes; fluoranthene; pyrene; all isomers of methyl- and dimethyl-pyrene; chrysene; all isomers of methyl- and dimethyl-chrysene. Only those hydrocarbons detected are shown in Figure 3.

#### qPCR Analysis of Bacterial and Archaeal 16S rRNA Genes

DNA was extracted from 0.25 g of thawed sediment samples with a DNeasy PowerSoil Kit (Qiagen), according to the manufacturer’s instructions. The primers used for quantification of archaeal 16S rRNA gene were 344f – ACGGGGYGCAGCAGGCGCGA (Raskin et al., 1994) and 915r – GTGCTCCCCCGCCAATTCCT (Stahl and Amann, 1991), and for bacterial 16S rRNA gene, 341f – CCTACGGGNGGCWGCAG, and 785r – GACTACHVGGGTATCTAATCC (Klindworth et al., 2013) were used. These primers have been successfully used to quantify archaeal (Huby et al., 2020) and bacterial (Clark et al., 2020) 16S rRNA gene abundances in environmental samples previously. Furthermore, inspection of standard curves showed that all assays produced satisfactory efficiency (85%) and *R*^2^ values (>0.99). All qPCR reactions were performed using a CFX384^TM^ Real-Time PCR Detection System (BioRad) using reagents, cycle conditions, and standards as previously described (McKew and Smith, 2015; Tatti et al., 2016).

#### Amplicon Sequencing and Bioinformatics

Amplicon libraries were prepared, as per Illumina instructions, by a 25-cycle (bacteria) and 31-cycle (archaea) PCR. PCR primers were the same as those used for qPCR but flanked with Illumina Nextera overhang sequences. A unique combination of Nextera XT Indices (Illumina) were added to PCR products from each sample, via an eight-cycle PCR. PCR products were quantified using PicoGreen and pooled in equimolar concentrations. Quantification of the amplicon libraries was determined via NEBNext^®^ Library Quant Kit for Illumina (New England BioLabs Inc.), prior to sequencing on the Illumina MiSeq^®^ platform, using a MiSeq^®^ 600 cycle v3 reagent kit and 20% PhiX sequencing control standard. Raw sequence data have been submitted to the European Nucleotide Archive database under accession number PRJEB33987. Sequence output from the Illumina MiSeq platform were analyzed within BioLinux (Field et al., 2006), using a bioinformatics pipeline as described by Dumbrell et al. (2016). Forward sequence reads were quality trimmed using Sickle (Joshi and Fass, 2011) prior to error correction within Spades (Nurk et al., 2013) using the BayesHammer algorithm (Nikolenko et al., 2013). The quality filter and error corrected sequence reads were dereplicated, sorted by abundance, and clustered into OTUs at the 97% sequence identity level via VSEARCH (Rognes et al., 2016). Singleton OTUs were discarded, as well as chimeras using reference based chimera checking with UCHIME (Edgar et al., 2011). Lastly, taxonomic assignment was conducted with the Ribosomal Database Project (RDP) classifier (Wang et al., 2007). Non-locus-specific, or artifactual, OTUs were discarded prior to statistical analyses, along with any OTUs that had <70% identity with any sequence in the RDP database.

#### Phylogenetic Analysis

The neighbor-joining protocol (Nei and Saitou, 1987) was used to infer the evolutionary history of partial 16S rRNA gene sequence Bacterial OTUs, aligned with known hydrocarbon-degrading bacteria and closest neighboring accessions using MUSCLE (Edgar, 2004). Bootstrapping analysis (1,000 iterations) was conducted to determine the percentage of time associated taxa clustered together in replicate trees (Felsenstein, 1985); only bootstrap values >70% are shown. Evolutionary distances, units in the number of base substitutions per site, were calculated with the use of Maximum Composite Likelihood protocol (Hanson and McElroy, 2015). Phylogenetic analyses were conducted in MEGA7.

#### Statistical Analysis

Prior to community analysis, sequence data were rarefied to the lowest library sequence value (8,093). Data were first tested for normality (Shapiro–Wilks test), those data which were normally distributed were tested for significance with ANOVAs or appropriate linear models. Non-normally distributed data were analyzed using appropriate GLMs (Generalized Linear Models) as follows. The relative abundance of OTUs or genera in relation to oil exposure, site, and sample month were modeled using multivariate negative binomial GLMs (Wang et al., 2010). Here, the number of sequences in each library was accounted for using an offset term, as described previously (Alzarhani et al., 2019). The abundance of archaeal and bacterial 16S rRNA gene copies was also modeled using negative binomial GLMs (Venables and Ripley, 2002). The significance of model terms was assessed via likelihood ratio tests. The EIHE (Lozada et al., 2014) was calculated using the script available at the ecolFudge GitHub page^1^ (Clark, 2019) and EIHE values modeled using Poisson GLMs. Correlations were performed using the Pearson’s correlation with an alpha of 0.05. All statistical analyses were carried out in R3.6.1 (R Development Core Team, 2011) using a variety of packages available through the references (Venables and Ripley, 2002; Becker et al., 2016; Auguie, 2017; Lenth, 2020). All plots were constructed using the (Wickham, 2010) and “patchwork” (Pedersen, 2019) R packages.

## Data Availability Statement

The datasets generated for this study can be found in the European Nucleotide Archive under accession number PRJEB33987.

## Author Contributions

GT, CW, TC, and BM carried out the sampling campaign. AM provided the country logistics and sampling support. GT and BG performed the molecular work. GT, PC, and FC performed the analytical chemistry. GT, BM, and DC performed the data and statistical analysis. GT wrote the manuscript, supplementary information, and produced all figures and tables. All authors reviewed the manuscript, supplementary information, figures, and tables. BM, TM, CW, TC, LH, and AM secured the funding. All authors contributed to the article and approved the submitted version.

## Conflict of Interest

The authors declare that the research was conducted in the absence of any commercial or financial relationships that could be construed as a potential conflict of interest.

## Footnotes

1 https://github.com/Dave-Clark/ecolFudge
