# Supplementary material for: Bacterial Community Legacy Effects Following the Agia Zoni II Oil-Spill, Greece
Source: Front Microbiol. 2020 Jul 17;11:1706. doi: 10.3389/fmicb.2020.01706 (PMC7379155; doi:10.3389/fmicb.2020.01706)
Supplement: Supplementary file 3 [file Table_1.DOCX]

Bacterial Community Legacy Effects following the Agia Zoni II Oil-Spill, Greece

Thomas G E^1^, Cameron, T C ^1^, Campo P^3^, Clark, D R^1^, Coulon F^3^, Gregson, B^1^, Hepburn, L J ^1^, McGenity T J^1^, Miliou A^2^, Whitby, C^1^, McKew B A*^1^

^1^ School of Life Sciences, University of Essex, Wivenhoe Park, Essex, CO4 3SQ, UK

^2^ Archipelagos Institute of Marine Conservation, P.O.42, Pythagorio, 83 103, Samos, Greece

^3^ Cranfield University, School of Water, Energy and Environment, Cranfield, MK43 0AL, UK

# *Correspondence:

*Dr Boyd A McKew, [boyd.mckew@essex.ac.uk](mailto:boyd.mckew@essex.ac.uk), +44 1206 873 010

**Tables 4 (Tables S3 and S4 are separate csv files)**

**Figures 5**


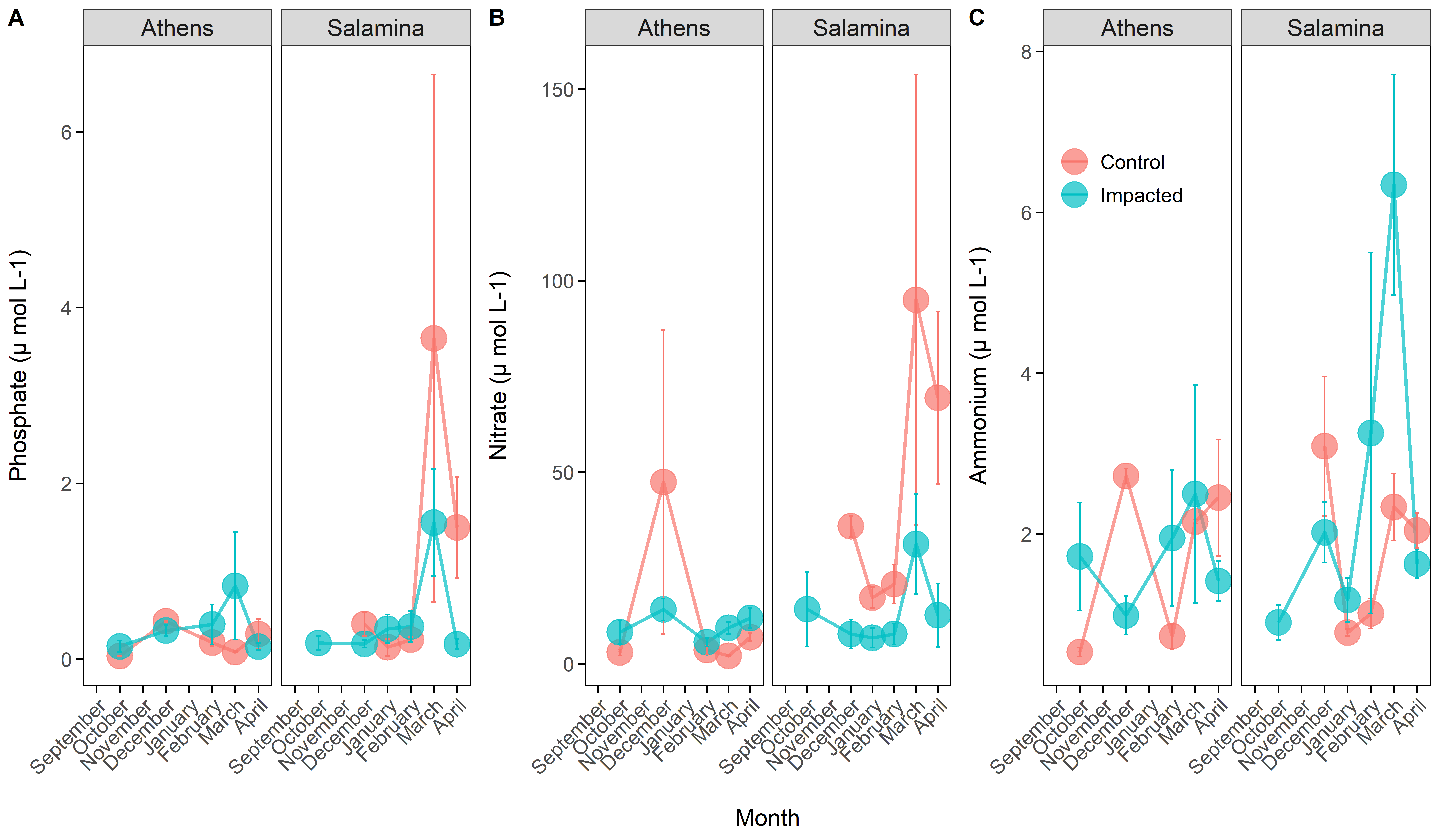


**Fig. S1**: Concentration (mean ± SE, *n* = 3) of phosphate (A), nitrate (B), and ammonium (C) in unimpacted (“Control”) and oil-impacted (“Impacted”) seawater samples from both the Athens Riviera and Salamina coastlines, sampled from October 2017 to April 2018 (seawater samples not taken in September 2017 at either site).


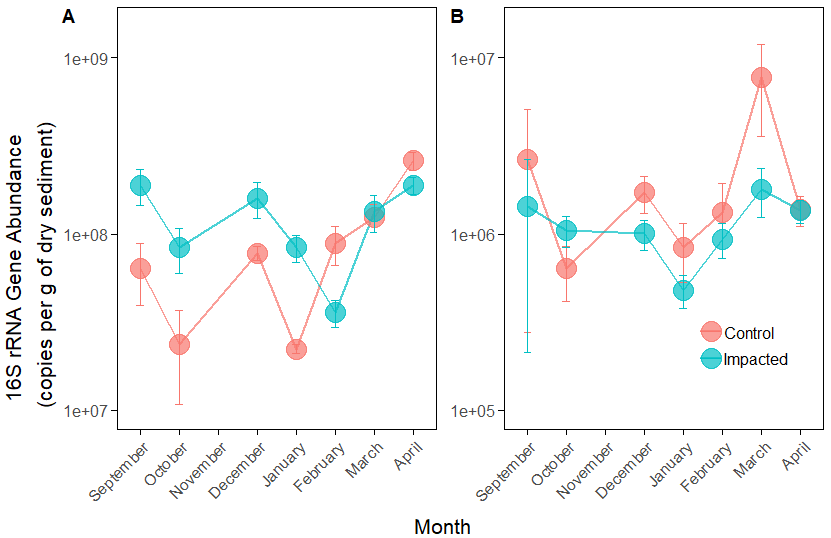


**Fig. S2**: A) bacterial, and B) archaeal, 16S rRNA gene abundance (mean ± SE, *n* = 6 (*n* = 3 for September as Athens only)) in unimpacted (“Control”) and oil-impacted (“Impacted”) sediments from both the Athens Riviera and Salamina coastlines, between September 2017 and April 2018.


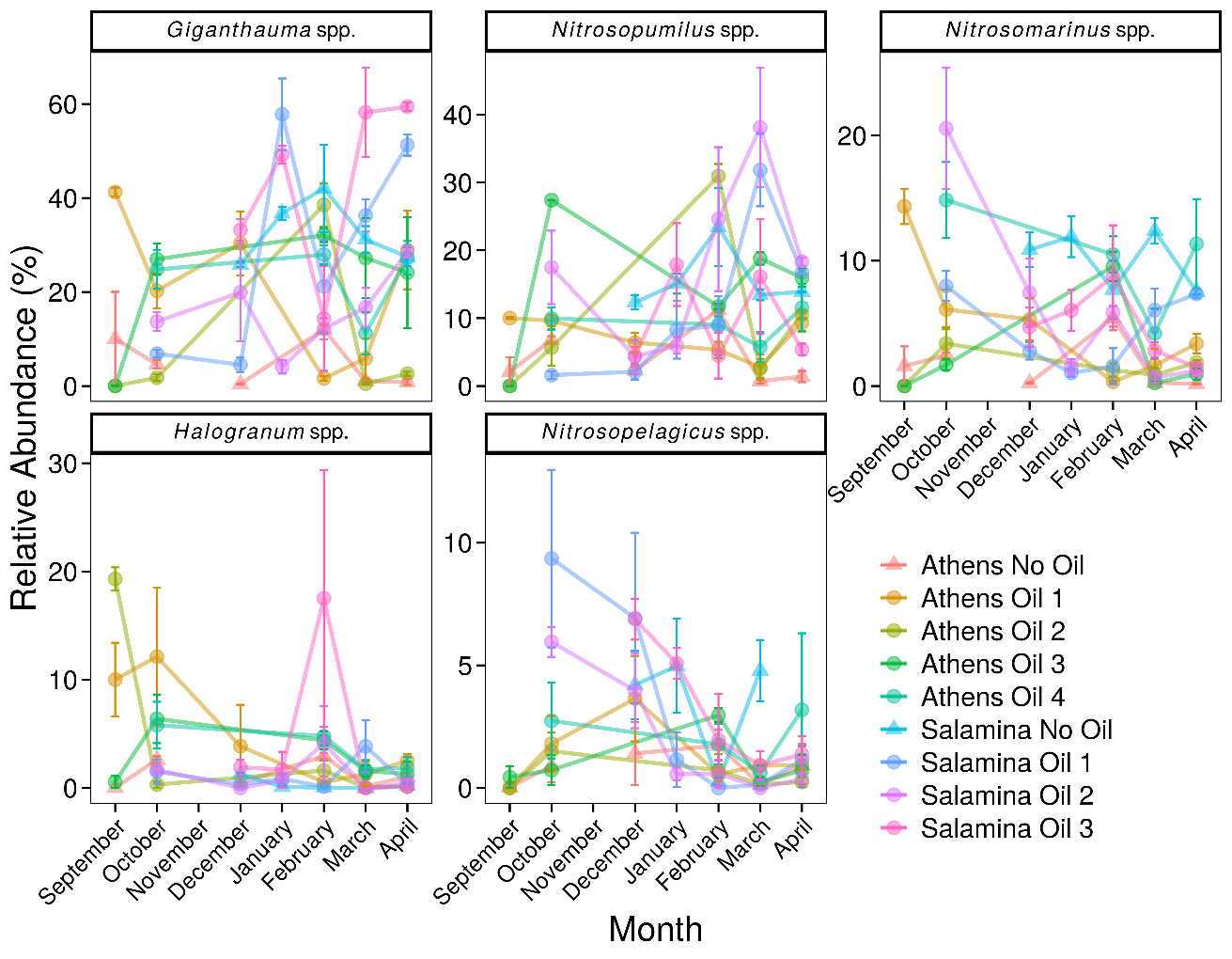


**Fig. S3**: Relative abundance (% of the archaeal community; mean ± SE, *n* = 3) of 16S rRNA gene OTUs assigned to the most abundant Archaea in sediments from control (Suffix “No Oil”) and oil-impacted (Suffix “Oil”) sites along the Athens Riviera and Salamina coastline, from September 2017 to April 2018.


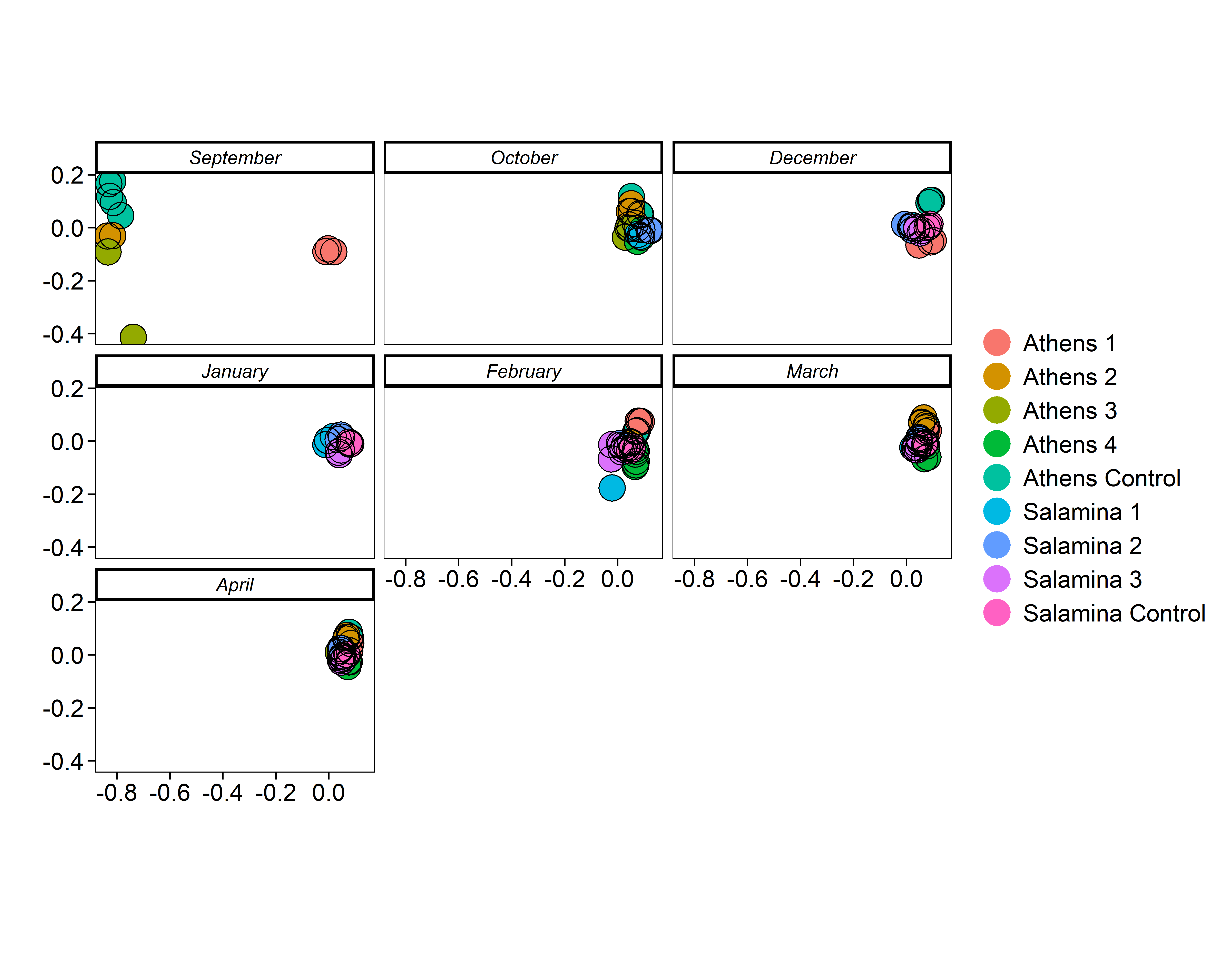


**Fig. S4**: NMDS (non-metric multidimensional scaling) ordination, based on clustered bacterial 16S rRNA OTUs at a 97% similarity threshold, displaying the effect of the Agia Zoni II oil-spill on bacterial community composition from September 2017 until April 2018.


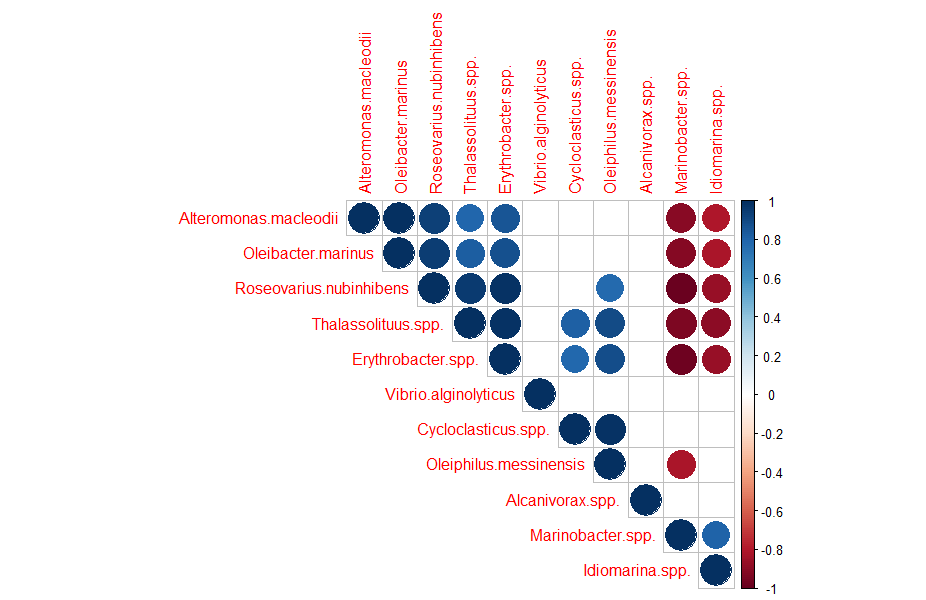


**Fig. S5**: Correlation matrix demonstrating positive (blue) or negative (red) relationships between different oil-degrading genera or species. Blank spaces refer to insignificant relationships.

**Table S1**: Site codes indicate the sampling location as referred to throughout the text and in figures, with exact coordinates (latitude and longitude) and area (either along the Athens Riviera, or, the Salamina coastline).

| **Site Code** | **Latitude** | **Longitude** | **Area** | **Response*** |
| --- | --- | --- | --- | --- |
| Athens No Oil | 37°43’1”N | 23°55’ E | Athens | No Oil Contamination |
| Athens Oil 1 | 37°51’3”N | 23°44’55”E | Athens | Manual Removal of Tar Balls |
| Athens Oil 2 | 37°51’39”N | 23°44’52”E | Athens | Flushing, Trenching, High-powered Washing, Sediment Removal |
| Athens Oil 3 | 37°51’54”N | 23°44’29”E | Athens | Flushing, Trenching, High-powered Washing, Sediment Removal |
| Athens Oil 4 | 37°52’4”N | 23°44’13”E | Athens | Flushing, Trenching, High-powered Washing, Sediment Removal |
| Salamina No Oil | 37°55’44”N | 23°32’19”E | Salamina | No Oil Contamination |
| Salamina Oil 1 | 37°56’31”N | 23°33’4”E | Salamina | Flushing, Trenching, High-powered Washing |
| Salamina Oil 2 | 37°56’34”N | 23°32’34”E | Salamina | Flushing, Trenching, High-powered Washing, Sediment Removal |
| Salamina Oil 3 | 37°56’25”N | 23°32’17”E | Salamina | High-powered Washing, Absorbents |

*Response refers to clean-up operations at the sampling location: Flushing (flushing of sediment with medium pressure water), Trenching (trenching, used in conjunction with flushing to collect oil), High-powered Washing (high-powered washing, to remove oil from hard surfaces), Sediment Removal (removal of coastal sediment, either washed and replace or disposed in landfill), Absorbents (use of absorbents to collect floating oil from the water surface).

**Table. S2**: Evidence demonstrating hydrocarbon degradation (alkane of PAH) and growth in oil-polluted marine environments (generally or specifically sandy coastal sediments) for certain genera of Bacteria (either OHCB or more metabolically versatile).

| **Genus** | **Hydrocarbon Degradation Metabolic Capability** | **Isolated Alkane Degraders** | **Polycyclic Aromatic Hydrocarbon Degraders** | **Evidence for Increased Abundance in Oiled Marine Environments^×^** | **Evidence for Increased Abundance Oiled Sandy Coastal Sediments^×^** |
| --- | --- | --- | --- | --- | --- |
| *Alcanivorax* spp. | OHCB* | **Yes** – (e.g. Yakimov *et al.*, 1998) | **No** (provision; may enhance PAH degradation utilising alkyl side-chains on methylated PAHs – e.g. Yuan *et al.*, 2015; Noh *et al.*, 2018) | **Yes** – (e.g.  Kasai *et al.*, 2001;  Röling *et al.*, 2004;  McKew *et al*., 2007;  Wang *et al.*, 2010;  Teramoto *et al.*, 2013;  Sanni *et al.*, 2015;  Lee *et al.*, 2017;  Liu *et al.*, 2019) | **Yes** – (e.g.  Kostka et al., 2011;  Newton *et al.*, 2013;  Lamendella et al., 2014;  Rodriguez-R *et al.*, 2015;  Curtis *et al.*, 2018;  Huettel *et al.*, 2018) |
| *Alteromonas* spp. | Versatile^±^ | **No** | **Yes** – (e.g.  *A. distincta (crude oil)*  Iwabuchi *et al.*, 2002; ***UD***  *A. naphthalenivorans* strain SN2  Jin *et al.*, 2011) | **Yes** – (e.g.  Kasai *et al.*, 2001;  Jin *et al*., 2011;  Jin *et al.*, 2012;  Teramoto *et al.*, 2013;  Linda *et al.*, 2018) | **Yes** - (e.g.  Newton *et al.*, 2013) |
| *Cycloclasticus* spp. | OHCB* | **No** (provision; *Cycloclasticus* sp. symbiont of *Bathymodiolus heckerae* – e.g. Rubin-Blum *et al.*, 2017) ***UD*** | **Yes** – (e.g. Dyksterhouse *et al.*, 1995) | **Yes** – (e.g.  Coulon *et al.*, 2007;  Dubinsky *et al.*, 2013;  Sanni *et al.*, 2015;  Lee *et al.*, 2017;  Liu *et al.*, 2017;  Linda *et al.*, 2018) | **Yes** – (e.g.  Geiselbrecht *et al.*, 1996;  Geiselbrecht *et al.*, 1998;  Röling *et al.*, 2002) |
| *Erythrobacter* spp. | Versatile^±^ | **Yes** – (e.g.  *E. flavus*  *E. seohaensis*  Harwati *et al.*, 2007) ***Both UD*** | **Yes** – (e.g.  *E. seohaensis*  *E. flavus*  Harwati *et al.*, 2007  Yuan *et al.*, 2015;  *E. atlanticus*  Zhuang *et al.*, 2015) ***All UD*** | **Yes** – (e.g.  McKew *et al*., 2007;  Liu and Liu, 2013;  Teramoto *et al.*, 2013;  Gao *et al.*, 2015) | **Yes** – (e.g.  Macnaughton *et al.*, 1999;  Röling *et al.*, 2002;  Jiménez *et al.*, 2007) |
| *Idiomarina* spp. | Versatile^±^ | **No** | **Yes** – (e.g.  *I. baltica* (crude oil)  Wang *et al.*, 2010; ***UD***  *I. seosinensis*  Yuan *et al.*, 2015;  Gomes *et al.*, 2018;  *I. piscisalsi*  Nzila *et al.*, 2018) | **Yes** – (e.g.  Röling *et al.*, 2004;  Wang *et al.*, 2010;  Albokari *et al.*, 2015) | **Yes** – (e.g.  Curtis *et al.*, 2018) |
| *Marinobacter* spp. | Versatile^±^ | **Yes** *– (e.g.*  *M. hydrocarbonoclasticus*  (Gauthier *et al.*, 1992)  *M. flavimaris* ***UD***  e.g. Gomes *et al.*, 2018) | **Yes** – (e.g.  *M. flavimaris*  *M. alkaliphilus*  Yuan *et al.*, 2015;  Gomes *et al.*, 2018) ***Both UD*** | **Yes** – (e.g.  Röling *et al.*, 2004;  McKew *et al*., 2007;  Teramoto *et al.*, 2013;  Albokari *et al*., 2015;  Sanni *et al.*, 2015;  Gao et al., 2015) | **Yes** – (e.g.  Kostka et al., 2011;  Newton *et al.*, 2013;  Lamendella et al., 2014;  Rodriguez-R *et al*., 2015;  Curtis *et al.*, 2018;  Huettel *et al.*, 2018) |
| *Oleibacter* spp. | OHCB* | **Yes** – (e.g.  Teramoto *et al.*, 2011) | **No** | **Yes** – (e.g.  Teramoto *et al.*, 2013;  Sanni *et al.*, 2015;  Liu *et al.*, 2017;  Liu *et al.*, 2019) | **No** |
| *Oleiphilus* spp. | OHCB* | **Yes** – (e.g.  Golyshin *et al.*, 2002;  Toshchakov *et al.*, 2017) | **No** | **Yes** – (e.g.  McKew *et al.*, 2007;  Yang *et al.*, 2016) | **No** |
| *Roseovarius* spp. | Versatile^±^ | **Yes** – (e.g.  *R. crassostreae*  (Harwati *et al.*, 2007)  ***UD*** | **Yes** – (e.g.  *R. crassostreae*  (Harwati *et al.*, 2007;  *R. mucosus*  Yuan *et al.*, 2015) ***Both UD*** | **Yes** – (e.g.  Vila *et al.*, 2010) | **Yes** – (e.g.  Gallego *et al.*, 2014;  Kappell *et al.*, 2014) |
| *Thalassolituus* spp. | OHCB^*^ | **Yes** – (e.g.  Yakimov *et al.*, 2004) | **No** | **Yes** – (e.g.  McKew *et al*., 2007;  Sanni *et al.*, 2015;  Lee *et al.*, 2017;  Liu *et al.*, 2019) | **No** |
| *Vibrio* spp. | Versatile^±^ | **Yes** – (e.g.  *V. alginolyticus* (Al-Awadhi *et al.*, 2012)  Diesel *V. alginolyticus* (Imron and Titah, 2018) | **Yes** – (e.g.  *V. cyclotrophicus* (Hedlund and Staley, 2001) ***UD***  *V. anguillarum* (Melcher *et al.*, 2002)  Diesel *V. alginolyticus* (Imron and Titah, 2018) | **Yes** – (e.g.  Teramoto *et al.*, 2013;  Sanni *et al.*, 2015) | **Yes** – (e.g.  Geiselbrecht *et al.*, 1996;  Rodriguez-R *et al*., 2015) |

* OHCB – refers to the so called “Obligate Hydrocarbonoclastic Bacteria” (Yakimov *et al.*, 2007)

^±^ Versatile – has the metabolic capabilities to grow on a diverse range of substrates

**^×^** These lists are by no means exhaustive but are examples of the growth of these genera in either *in situ* or *ex situ* oil-based systems.

**UD** – this species was not detected in this study

**References**

Al-Awadhi, H., Dashti, N., Kansour, M., Sorkhoh, N., and Radwan, S. (2012) Hydrocarbon-utilizing bacteria associated with biofouling materials from offshore waters of the Arabian Gulf. *Int Biodeterior Biodegrad* **69**: 10–16.

Albokari, M., Mashhour, I., Alshehri, M., Boothman, C., and Al-Enezi, M. (2015) Characterization of microbial communities in heavy crude oil from Saudi Arabia. *Ann Microbiol* **65**: 95–104.

Coulon, F., McKew, B.A., Osborn, A.M., McGenity, T.J., and Timmis, K.N. (2007) Effects of temperature and biostimulation on oil-degrading microbial communities in temperate estuarine waters. *Environ Microbiol* **9**: 177–186.

Curtis, D., Elango, V., Collins, A.W., Rodrigue, M., and Pardue, J.H. (2018) Transport of crude oil and associated microbial populations by washover events on coastal headland beaches. *Mar Pollut Bull* **130**: 229–239.

Dubinsky, E.A., Conrad, M.E., Chakraborty, R., Bill, M., Borglin, S.E., Hollibaugh, J.T., et al. (2013) Succession of hydrocarbon-degrading bacteria in the aftermath of the deepwater horizon oil spill in the gulf of Mexico. *Environ Sci Technol* **47**: 10860–10867.

Dyksterhouse, S.E., GRAY, J.P., HERWIG, R.P., LARA, J.C., and STALEY, J.T. (1995) Cycloclasticus pugetti gen. nov., sp. nov., an aromatic hydrocarbon-degrading bacterium from marine sediments. *Int J Syst Bacteriol* **45**: 116-123.

Gallego, S., Vila, J., Tauler, M., Nieto, J.M., Breugelmans, P., Springael, D., and Grifoll, M. (2014) Community structure and PAH ring-hydroxylating dioxygenase genes of a marine pyrene-degrading microbial consortium. *Biodegradation* **25**: 543–556.

Gao, X., Gao, W., Cui, Z., Han, B., Yang, P., Sun, C., and Zheng, L. (2015) Biodiversity and degradation potential of oil-degrading bacteria isolated from deep-sea sediments of South Mid-Atlantic Ridge. *Mar Pollut Bull* **97**: 373–380.

Gauthier, M.J., Lafay, B., Christen, R., Fernandez, L., Acquaviva, M., Bonin, P., and Bertrand, J.-C. (1992) Marinobacter hydrocarbonoclasticus gen. nov., sp. nov., a New, Extremely Halotolerant, Hydrocarbon-Degrading Marine Bacterium. *Int J Syst Bacteriol* **42**: 568–576.

Geiselbrecht, A.D., Hedlund, B.P., Tichi, M.A., and Staley, J.T. (1998) Isolation of marine polycyclic aromatic hydrocarbon (PAH)-degrading Cycloclasticus strains from the Gulf of Mexico and comparison of their PAH degradation ability with that of Puget Sound Cycloclasticus strains. *Appl Environ Microbiol* **64**: 4703–4710.

Geiselbrecht, A.D., Herwig, R.P., Deming, J.W., and Staley, J.T. (1996) Enumeration and phylogenetic analysis of polycyclic aromatic hydrocarbon-degrading marine bacteria from Puget Sound sediments. *Appl Environ Microbiol* **62**: 3344–3349.

Golyshin, P.N., Chernikova, Ta.N., Abraham, W.R., Lünsdorf, H., Timmis, K.N., and Yakimov, M.M. (2002) Oleiphilaceae fam. nov., to include Oleiphilus messinensis gen. nov., sp. nov., a novel marine bacterium that obligately utilizes hydrocarbons. *Int J Syst Evol Microbiol* **52**: 901–911.

Gomes, M.B., Gonzales-Limache, E.E., Sousa, S.T.P., Dellagnezze, B.M., Sartoratto, A., Silva, L.C.F., et al. (2018) Exploring the potential of halophilic bacteria from oil terminal environments for biosurfactant production and hydrocarbon degradation under high-salinity conditions. *Int Biodeterior Biodegrad* **126**: 231–242.

Harwati, T.U., Kasai, Y., Kodama, Y., Susilaningsih, D., and Watanabe, K. (2007) Characterization of Diverse Hydrocarbon-Degrading Bacteria Isolated from Indonesian Seawater. *Microbes Environ* **22**: 412–415.

Hedlund, B.P. and Staley, J.T. (2001) Vibrio cyclotrophicus sp. nov., a polycyclic aromatic hydrocarbon (PAH)-degrading marine bacterium. *Int J Syst Evol Microbiol* **51**: 61–66.

Huettel, M., Overholt, W.A., Kostka, J.E., Hagan, C., Kaba, J., Wells, W.B., and Dudley, S. (2018) Degradation of Deepwater Horizon oil buried in a Florida beach influenced by tidal pumping. *Mar Pollut Bull* **126**: 488–500.

Imron, M.F. and Titah, H.S. (2018) Optimization of diesel biodegradation by vibrio alginolyticus using Box-Behnken design. *Environ Eng Res* **23**: 374–382.

Iwabuchi, N., Sunairi, M., Urai, M., Itoh, C., Anzai, H., Nakajima, M., and Harayama, S. (2002) Extracellular polysaccharides of Rhodococcus rhodochrous S-2 stimulate the degradation of aromatic components in crude oil by indigenous marine bacteria. *Appl Environ Microbiol* **68**: 2337–2343.

Jiménez, N., Viñas, M., Bayona, J.M., Albaiges, J., and Solanas, A.M. (2007) The Prestige oil spill: Bacterial community dynamics during a field biostimulation assay. *Appl Microbiol Biotechnol* **77**: 935–945.

Jin, H.M., Jeong, H., Moon, E.J., Math, R.K., Lee, K., Kim, H.J., et al. (2011) Complete genome sequence of the polycyclic aromatic hydrocarbon-degrading bacterium Alteromonas sp. strain SN2. *J Bacteriol* **193**: 4292–4293.

Jin, H.M., Kim, J.M., Lee, H.J., Madsen, E.L., and Jeon, C.O. (2012) Alteromonas as a key agent of polycyclic aromatic hydrocarbon biodegradation in crude oil-contaminated coastal sediment. *Environ Sci Technol* **46**: 7731–7740.

Kappell, A.D., Wei, Y., Newton, R.J., van Nostrand, J.D., Zhou, J., McLellan, S.L., and Hristova, K.R. (2014) The polycyclic aromatic hydrocarbon degradation potential of Gulf of Mexico native coastal microbial communities after the Deepwater Horizon oil spill. *Front Microbiol* **5**: 1–13.

Kasai, Y., Kishira, H., Syutsubo, K., and Harayama, S. (2001) Molecular detection of marine bacterial populations on beaches contaminated by the Nakhodka tanker oil-spill accident. *Environ Microbiol*.

Lee, J., Han, I., Kang, B.R., Kim, S.H., Sul, W.J., and Lee, T.K. (2017) Degradation of crude oil in a contaminated tidal flat area and the resilience of bacterial community. *Mar Pollut Bull* **114**: 296–301.

Linda, A., Hernado, P.B., and Yuewen, D. (2018) Response of microbial communities to oil spill in the Gulf of Mexico: A review. *African J Microbiol Res* **12**: 536–545.

Liu, J., Bacosa, H.P., and Liu, Z. (2017) Potential environmental factors affecting oil-degrading bacterial populations in deep and surface waters of the Northern Gulf of Mexico. *Front Microbiol* **7**: 1–14.

Liu, J., Zheng, Y., Lin, H., Wang, X., Li, M., Liu, Y., et al. (2019) Proliferation of hydrocarbon-degrading microbes at the bottom of the Mariana Trench. *Microbiome* **7**: 1–13.

Liu, Z. and Liu, J. (2013) Evaluating bacterial community structures in oil collected from the sea surface and sediment in the northern Gulf of Mexico after the Deepwater Horizon oil spill. *Microbiologyopen* **2**: 492–504.

Macnaughton, S.J., Stephen, J.R., Venosa, A.D., Davis, G.A., Chang, Y.J., and White, D.C. (1999) Microbial population changes during bioremediation of an experimental oil spill. *Appl Environ Microbiol*.

McKew, B.A., Coulon, F., Osborn, A.M., Timmis, K.N., and McGenity, T.J. (2007) Determining the identity and roles of oil-metabolizing marine bacteria from the Thames estuary, UK. *Environ Microbiol* **9**: 165–176.

Melcher, R.J., Apitz, S.E., and Hemmingsen, B.B. (2002) Impact of irradiation and polycyclic aromatic hydrocarbon spiking on microbial populations in marine sediment for future aging and biodegradability studies. *Appl Environ Microbiol*.

Newton, R.J., Huse, S.M., Morrison, H.G., Peake, C.S., Sogin, M.L., and McLellan, S.L. (2013) Shifts in the Microbial Community Composition of Gulf Coast Beaches Following Beach Oiling. *PLoS One* **8**: 1–13.

Noh, J., Kim, H., Lee, C., Yoon, S.J., Chu, S., Kwon, B.O., et al. (2018) Bioaccumulation of Polycyclic Aromatic Hydrocarbons (PAHs) by the Marine Clam, Mactra veneriformis, Chronically Exposed to Oil-Suspended Particulate Matter Aggregates. *Environ Sci Technol* **52**: 7910–7920.

Nzila, A., Jung, B.K., Kim, M.C., Ibal, J.C., Budiyanto, F., Musa, M.M., et al. (2018) Complete genome sequence of the polycyclic aromatic hydrocarbons biodegrading bacterium Idiomarina piscisalsi strain 10PY1A isolated from oil-contaminated soil. *Korean J Microbiol* **54**: 289–292.

Rodriguez-R, L.M., Overholt, W.A., Hagan, C., Huettel, M., Kostka, J.E., and Konstantinidis, K.T. (2015) Microbial community successional patterns in beach sands impacted by the Deepwater Horizon oil spill. *ISME J* **9**: 1928–1940.

Röling, W.F.M., Milner, M.G., Jones, D.M., Fratepietro, F., Swannell, R.P.J., Daniel, F., and Head, I.M. (2004) Bacterial community dynamics and hydrocarbon degradation during a field-scale evaluation of bioremediation on a mudflat beach contaminated with buried oil. *Appl Environ Microbiol* **70**: 2603–2613.

Röling, W.F.M., Milner, M.G., Jones, D.M., Lee, K., Daniel, F., Swannell, R.J.P., and Head, I.M. (2002) Robust hydrocarbon degradation and dynamics of bacterial communities during nutrient-enhanced oil spill bioremediation. *Appl Environ Microbiol* **68**: 5537–5548.

Rubin-Blum, M., Antony, C.P., Borowski, C., Sayavedra, L., Pape, T., Sahling, H., et al. (2017) Short-chain alkanes fuel mussel and sponge Cycloclasticus symbionts from deep-sea gas and oil seeps. *Nat Microbiol* **2**:.

Sanni, G.O., Coulon, F., and McGenity, T.J. (2015) Dynamics and distribution of bacterial and archaeal communities in oil-contaminated temperate coastal mudflat mesocosms. *Environ Sci Pollut Res* **22**: 15230–15247.

Teramoto, M., Ohuchi, M., Hatmanti, A., Darmayati, Y., Widyastuti, Y., Harayama, S., and Fukunaga, Y. (2011) Oleibacter marinus gen. nov., sp. nov., a bacterium that degrades petroleum aliphatic hydrocarbons in a tropical marine environment. *Int J Syst Evol Microbiol* **61**: 375–380.

Teramoto, M., Queck, S.Y., and Ohnishi, K. (2013) Specialized Hydrocarbonoclastic Bacteria Prevailing in Seawater around a Port in the Strait of Malacca. *PLoS One* **8**: 2–8.

Toshchakov, S. V., Korzhenkov, A.A., Chernikova, T.N., Ferrer, M., Golyshina, O. V., Yakimov, M.M., and Golyshin, P.N. (2017) The genome analysis of Oleiphilus messinensis ME102 (DSM 13489T) reveals backgrounds of its obligate alkane-devouring marine lifestyle. *Mar Genomics* **36**: 41–47.

Vila, J., Nieto, J.M., Mertens, J., Springael, D., and Grifoll, M. (2010) Microbial community structure of a heavy fuel oil-degrading marine consortium: Linking microbial dynamics with polycyclic aromatic hydrocarbon utilization. *FEMS Microbiol Ecol* **73**: 349–362.

Wang, L., Wang, W., Lai, Q., and Shao, Z. (2010) Gene diversity of CYP153A and AlkB alkane hydroxylases in oil-degrading bacteria isolated from the Atlantic Ocean. *Environ Microbiol* **12**: 1230–1242.

Wang, W., Wang, L., and Shao, Z. (2010) Diversity and Abundance of Oil-Degrading Bacteria and Alkane Hydroxylase (alkB) Genes in the Subtropical Seawater of Xiamen Island. *Microb Ecol* **60**: 429–439.

Yakimov, M.M., Giuliano, L., Denaro, R., Crisafi, E., Chernikova, T.N., Abraham, W.R., et al. (2004) Thalassolituus oleivorans gen. nov., sp. nov., a novel marine bacterium that obligately utilizes hydrocarbons. *Int J Syst Evol Microbiol* **54**: 141–148.

Yakimov, M.M., Golyshin, P.N., Lang, S., Moore, E.R.B., Abraham, W.-R., Lunsdorf, H., and Timmis, K.N. (1998) Alcanivorax borkumensis gen. nov., sp. nov., a new, hydrocarbon-degrading and surfactant-producing marine bacterium. *Int J Syst Bacteriol* **48**: 339–348.

Yakimov, M.M., Timmis, K.N., and Golyshin, P.N. (2007) Obligate oil-degrading marine bacteria. *Curr Opin Biotechnol* **18**: 257–266.

Yang, T., Nigro, L.M., Gutierrez, T., D’Ambrosio, L., Joye, S.B., Highsmith, R., and Teske, A. (2016) Pulsed blooms and persistent oil-degrading bacterial populations in the water column during and after the Deepwater Horizon blowout. *Deep Res Part II Top Stud Oceanogr* **129**: 282–291.

Yuan, J., Lai, Q., Sun, F., Zheng, T., and Shao, Z. (2015) The diversity of PAH-degrading bacteria in a deep-sea water column above the southwest Indian ridge. *Front Microbiol* **6**: 1–12.

Zhuang, L., Liu, Y., Wang, L., Wang, W., and Shao, Z. (2015) Erythrobacter atlanticus sp. nov., a bacterium from ocean sediment able to degrade polycyclic aromatic hydrocarbons. *Int J Syst Evol Microbiol* **65**: 3714–3719.
